# Supplementary material for: Patterns of Intron Gain and Loss in Fungi
Source: PLoS Biol. 2004 Nov 30;2(12):e422. doi: 10.1371/journal.pbio.0020422 (PMC532390; doi:10.1371/journal.pbio.0020422)
Supplement: Table S1 — Also available at http://genes.mit.edu/NielsenEtAl/. (4.3 MB ZIP). [file pbio.0020422.st001.zip › NielsenEtAl/html/824.html]

AN1263.1.NCU07930.1.MG05155.1.FG05615.1


```
 CLUSTAL W (1.82) Multiple Sequence Alignments - Introns Inserted


Sequence 1: NCU07930.1	449 aa
Sequence 2: MG05155.1	449 aa
Sequence 3: FG05615.1	328 aa
Sequence 4: AN1263.1	449 aa
Alignment Length: 449 aa
Number Identitical Residues: 265 aa
Alignment Score (without introns) 12644


MG05155.1 	MAAPAHKFKVADL~SLAAFGRKEIELAENEMPGLMQTREKYAADQPLKGARIAGCLHMT~
NCU07930.1	MSAPAHKFKVADL~SLAAFGRKEIELAENEMPGLMATRKKYAADQPLKGARIAGCLHMT~
FG05615.1 	MSAPAHKFKVADL~SLAAFGRKEIELAENEMPGLMQTRAKYAADQPLAGARIAGCLHMT~
AN1263.1  	MAAPAQKFKVADI0SLAAFGRREIELAEIEMPGLMAIRAKYGPAQPLKGARIAGCLHMT1
          	*:***:******: *******:****** ******  * **.. *** *********** 

MG05155.1 	IQTAVLIETLTALGAEVTWTSCNIFSTQDHAAAAIAAAGVPV~FAWKGETEEEYNWCLEQ
NCU07930.1	IQTAVLIETLTALGAEVTWSSCNIFSTQDHAAAAIAAAGVPV~FAWKGETEEEYQWCLEQ
FG05615.1 	IQTAVLIETLTALGAEVTWTSCNIFSTQDHAAAAIAAAGVPV~FAWKGETDEEYNWCLEQ
AN1263.1  	IQTAVLIETLTALGAEVTWTSCNIFSTQDHAAAAIAAAGVPV2FAWKGETEEEYNWCLEQ
          	*******************:********************** *******:***:*****

MG05155.1 	QLLAFKDGKKLNLILDDGGDLTHLVHDKYPEMLADCYGVSEETTTGVHHLYKMLKEGKLL
NCU07930.1	QLIAFKDNKKLNLILDDGGDLTHLVHTKYPEMLEDCFGVSEETTTGVHHLYRMLKEGKLL
FG05615.1 	QLTAFKDNKKLNLILDDGGDLTTLVHQKYPEMLKDCFGVSEETTTGVHHLYRMLRDNKLL
AN1263.1  	QLSAFKDGQKLNLILDDGGDLTSLVHTKYPEQLQGCYGLSEETTTGVHHLYRMLKEGKLL
          	** ****.:************* *** **** * .*:*:************:**::.***

MG05155.1 	VPAINVNDSVTKSKFDNLYGCRESLVDGIKRATDVMIAGKIAVVAGYGDVGKGCAMALHG
NCU07930.1	VPAINVNDSVTKSKFDNLYGCRESLVDGIKRATDVMIAGKIAVVAGFGDVGKGCAMALSG
FG05615.1 	VPAINVNDSVTKSKFDNLYGCRESLVDGIKRATDVMIAGKVAVVAGFGDVGKGCAMALHG
AN1263.1  	VPAINVNDSVTKSKFDNLYGCRESLIDGIKRATDVMIAGKVAVVAGFGDVGKGCAQALHT
          	*************************:**************:*****:******** **  

MG05155.1 	MGARVIVTEIDPINALQAAMAGFQVTTMEKAASVGQIFVTTTGCRDILVGKHFEAMPNDA
NCU07930.1	MGARVIVTEVDPINALQAAMAGYQVTTMEKAAPLGQIFVTTTGCRDILVGKHFEVMPNDA
FG05615.1 	MGARVIVTEIDPINALQAAMAGYQVTTMEKAAKFGQIFVTTTGCRDILTGEHFEAMPNDA
AN1263.1  	MGARVLVTEIDPINALQAAVQGYEVVTMEEAAPLGQIFVTTTGCRDILVGKHFEVMRNDA
          	*****:***:*********: *::*.***:** .**************.*:***.* ***

MG05155.1 	IVCN~IGHFDIEIDVAWLKANAKSVQNIKPQVDRFLMANGRHIILLAEGRLVNLGCATGH
NCU07930.1	IVCN~IGHFDVEIDVAWLKANAASVQNIKPQVDRFLMKNGRHIILLAEGRLVNLGCATGH
FG05615.1 	IVCN~IGHFDIEIDVCLAQE-------------------ERHFCPEHQG--------PG-
AN1263.1  	IVCN1IGHFDIEIDVAWLKANAKSVQNIKPQVDRYLMPNGRHIILLAEGRLVNLGCATGH
          	**** *****:****.  : .: : .. ... .     . **:    :*   . ..:.* 

MG05155.1 	SSFVMSCSFTNQVLAQIMLYKNNDAAFGQKYVEFAKSGKLEKKVYVLPKILDEEVAKLHL
NCU07930.1	SSFVMSCSFTNQVLAQIMLYKANDEAFSNKYVEFGKSGKLEKKVYVLPKILDEEVARLHL
FG05615.1 	------------------------------------------------------------
AN1263.1  	SSFVMSCSFSNQVLAQIALFKAEDKDFGQKYPEFGTSGKKPVGVYVLPKILDEQVAYLHL
          	::   :.: :..  :.    . ..   ...  . ..:..        ..  ... :    

MG05155.1 	AHCNVELSTLTPVQAEYLSLPAEGPYKPEH~YRY
NCU07930.1	DHCNVELTQLSDVQAEYLGLATEGPYKSDH2YRY
FG05615.1 	------------------------------~---
AN1263.1  	SHVNAKLSTLTPVQAEYLGLDAKGPYKADH~YRY
          	   . . :  :  .:.  .  :... ...
```
